# Supercritical Fluid Extract of *Angelica sinensis* and *Zingiber officinale* Roscoe Ameliorates TNBS-Induced Colitis in Rats

**DOI:** 10.3390/ijms20153816

**Published:** 2019-08-05

**Authors:** Jia Liu, Ling Yu, Nuolan Mo, Hai Lan, Yan Zhang, Xin Liu, Qing Wu

**Affiliations:** School of Chinese Materia Medica, Beijing University of Chinese Medicine, Beijing 102488, China

**Keywords:** *Angelica sinensis*, *Zingiber officinale* Roscoe, inflammatory bowel disease, supercritical fluid extract, anti-inflammatory

## Abstract

Inflammatory bowel disease (IBD) is a worldwide healthcare problem calling for the development of new therapeutic drugs. *Angelica sinensis* and *Zingiber officinale* Roscoe are two common dietetic Chinese herbs, which are traditionally used for complementary treatment of gastrointestinal disorders. As bioactive constituents, volatile and pungent substances of these two herbs could be effectively extracted together by supercritical fluid extraction. In this study, the supercritical fluid extract of *Angelica sinensis* and *Zingiber officinale* Roscoe (AZ-SFE) was obtained by an optimized extraction process and it was chemically characterized. The anti-inflammatory effect and underlying mechanism of AZ-SFE were evaluated in a lipopolysaccharide (LPS)-induced RAW264.7 cell model and a 2, 4, 6-trinitrobenzenesulfonic acid (TNBS)-induced colitis rat model. AZ-SFE notably inhibited the production of NO in LPS-stimulated macrophages, and it inhibited the proliferation of Concanavalin A (Con A)-induced splenocytes with suppression of the Th1 immune response. In vivo, the study demonstrated that AZ-SFE significantly alleviated disease activity, colonic shortening, macroscopic damage and histological injury of TNBS-treated rats with reduction of oxidative stress, suppression of inflammatory cytokines, and modulation of hepcidin and serum iron. These findings suggested that AZ-SFE may be a promising supplement for current IBD therapy.

## 1. Introduction

Inflammatory bowel disease (IBD), including ulcerative colitis (UC) and Crohn’s disease (CD), is a worldwide healthcare problem characterized by chronic and recurring inflammation in the intestinal tract [1,2]. Up to date, the pathogenesis of IBD has not been clearly clarified, which may be relevant to genetic susceptibility, environmental factors, and intestinal flora, subsequently affecting the immune response, resulting in inflammation [2,3]. Also, oxidative stress could activate inflammatory mediators, which should not be neglected [4]. More seriously, it has been widely accepted that IBD is an important risk factor for the development of colorectal cancer [5]. Current treatments of IBD mainly rely on the usage of aminosalicylic acids, corticosteroids, thiopurines, and biologic drugs [6]. However, these drugs are often expensive and associated with side effects or undesirable clinical efficacy [7]. Therefore, investigation of novel therapeutic drugs for IBD treatment is needed.

Finding potential therapeutic candidates from natural products based on food and complementary and alternative medicine has become attractive, due to their long history of use. In recent years, a series of herbal compounds with anti-inflammatory effects have been reported with the possibility of treatment for IBD, such as gingerol, curcumin, resveratrol and anthocyanins [8,9,10]. Plant-derived substances capable of ameliorating IBD symptoms could potentially enlarge the scope of drug candidates.

*Angelica sinensis* and *Zingiber officinale* Roscoe are two common dietetic Chinese herbs which are traditionally applied for complementary treatment of gastrointestinal disorders [11]. The major components of them in common are volatile oils, which are a group of constituents with various bioactivities, including anti-inflammatory, anti-oxidant, and anti-cancer activities [12,13]. Previous studies proved the anti-inflammatory effects of *Angelica sinensis*’s volatile oils on acute inflammation model rats [14], and essential oils from *Zingiber officinale* Roscoe could inhibit leukocyte migration to exert an anti-inflammatory effect [15]. In addition, gingerols and their derivatives, as important constituents in *Zingiber officinale* Roscoe, are non-volatile pungent compounds with multiple functions, which have been reviewed by Butt et al. [16]. Specifically, gingerols showed protective effects on IBD mice and rats by reducing cytokines and adjusting oxidative stress [17,18]. A random clinical trial indicated that the supplementation of ginger powder orally could improve treatment of UC patients with significant reduction of malonic dialdehyde (MDA) [19]. Furthermore, our previous study proved the chemo-preventive potential of *Angelica sinensis* on an Azoxymethane/Dextran sodium sulphate induced colorectal cancer model [20]. Taken together, the combination of *Angelica sinensis* and *Zingiber officinale* Roscoe have potential for the treatment of IBD.

It is well known that choosing the proper extract method is necessary for the improvement of extract quality and yield. Supercritical fluid extraction is an efficient and environmentally friendly method to extract non-polar constituents from vegetal sources, especially for those thermal unstable compounds [21]. It is a great choice to extract potential anti-colitis components from the two above mentioned herbs by this method.

Many animal models have been applied to study IBD, such as chemical-induced colitis models, models of spontaneous colitis, and genetically engineered animal models. Among them, chemically induced models are most commonly used due to their reproducibility and ease of operation [22]. The chemical substance 2,4,6-trinitrobenzenesulfonic acid (TNBS) is commonly used for induction of IBD, and many previous studies used a TNBS-induced rodent model to evaluate the therapeutic potency of various substances on IBD [7,23]. 

Therefore, the first aim of this study was to obtain the supercritical fluid extract of *Angelica sinensis* and *Zingiber officinale* Roscoe (AZ-SFE) after optimization of extraction process parameters by orthogonal experimental design and to characterize it. In addition, the anti-inflammatory potential of AZ-SFE in vitro was investigated by detecting NO production on lipopolysaccharide (LPS)-induced RAW264.7 cells. Moreover, the anti-colitis effects of AZ-SFE on a TNBS-induced colitis rat model were evaluated through different biological activities.

## 2. Results

### 2.1. Qualitative Analysis of AZ-SFE Based on GC/MS Analysis

The qualitative analysis of AZ-SFE was based on NIST14 Standard Reference Database and literature references. As shown in Table 1, 34 compounds in AZ-SFE were identified, accounting for more than 95% of the total peak area. The component with the largest normalized peak area was Z-ligustilide (30.90%), followed by 6-gingerol (16.08%). Considering the lack of standard substances for other major compounds, such as zingiberene and α-farnesene, their contents were not detected in subsequent studies.

### 2.2. Optimization of Extraction Process of AZ-SFE by Orthogonal Experimental Design

**Figure 1:**
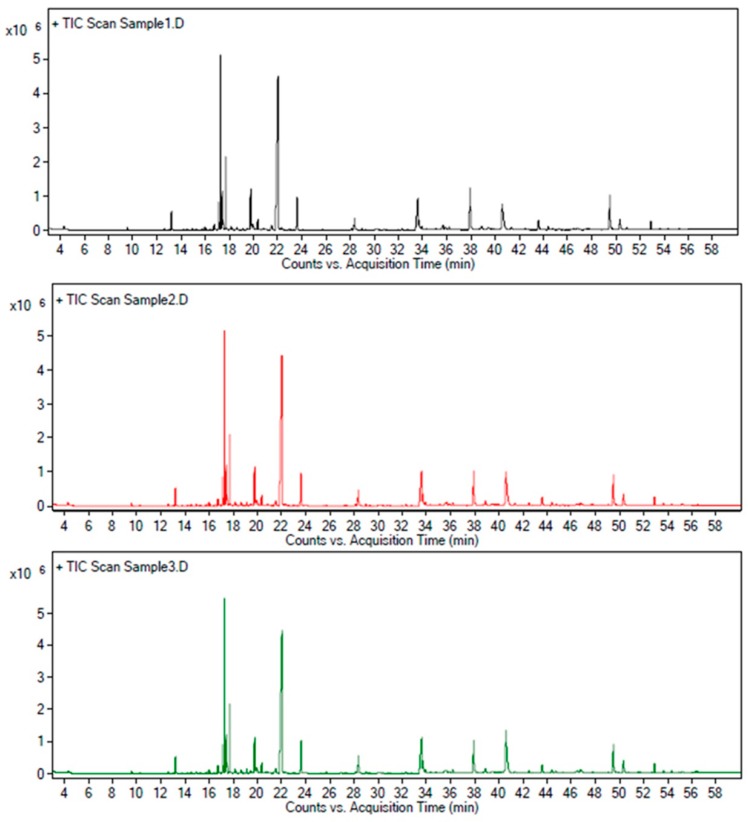
GC/MS total ion chromatogram of three batches of AZ-SFE extracted by optimized parameters.

The orthogonal experimental design was conducted for the optimization of three parameters (extraction pressure, temperature, and time) in the extraction process of AZ-SFE. As indicated in Table 2, experiment number 6 (55 °C, 30 MPa, extraction for 1 h) possessed the highest colligation score among experimental results. The importance ranking of three factors was: B (temperature) > A (pressure) > C (time), based on R value. Variance analysis indicated that extraction temperature was significant, while extraction pressure and time were non-significant (Table 3). Taken together, optimum extraction parameters were as follows: Extraction pressure at 30 MPa, extraction temperature at 55 °C, extraction for 1 h. For validation of the stability of the process, the experiment was conducted at optimal condition three times, and extracts obtained were analyzed by HPLC and GC/MS (Figure 1). The average yield of AZ-SFE was 2.88 ± 0.10%, content of ligustilide was 14.20 ± 0.11% (m/m), and content of 6-gingerol was 8.19 ± 0.19% (*m/m*). Particularly, more than 95% of ligustilide and 6-gingerol in plant materials were extracted under optimized conditions. Furthermore, the normalized area of each peak was stable with RSD less than 5% by GC/MS analysis. Hence, AZ-SFE with stable quality was acquired, which could be further studied.

### 2.3. Effects of AZ-SFE and Major Components on Cell Viability of RAW264.7 Cells

**Figure 2:**
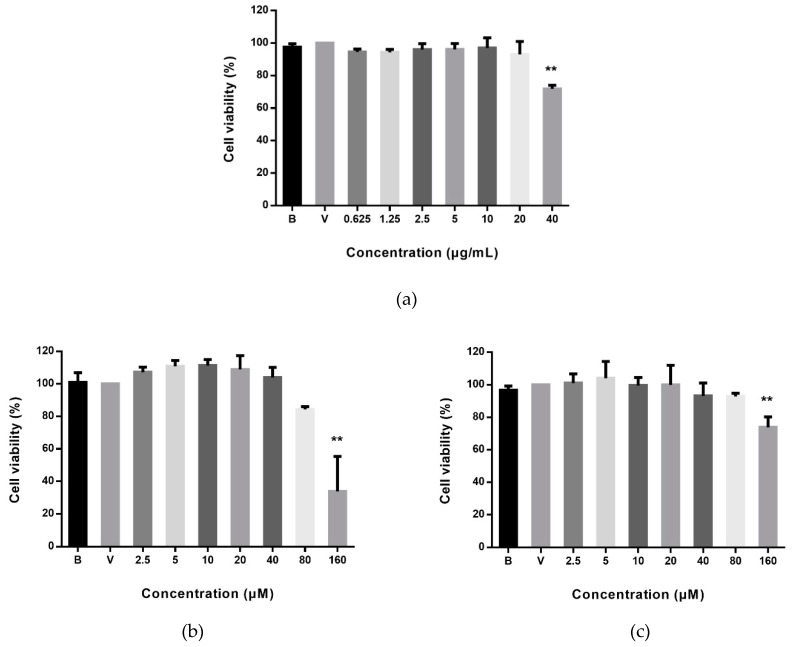
Cell viability of RAW264.7 cells after 24 h treatment of (**a**) AZ-SFE, (**b**) ligustilide, and (**c**) 6-gingerol detected by MTT assay. B represented medium group and V represented vehicle control group. Data are expressed as mean ± SD (*n* = 5) of three independent experiments. ** *p* < 0.01 versus vehicle control group.

As shown in Figure 2, only the highest dose (40 μg/mL) of AZ-SFE detected significantly reduced cell viability of RAW264.7 cells, by approximately 30%. AZ-SFE ranging from 0–20 μg/mL without cytotoxicity was studied further. Meanwhile, the effects of ligustilide and 6-gingerol on cell viability of RAW264.7 cells were assessed. Results indicated that there was no significant cell proliferation inhibition (cell viability < 80%) at the concentrations of 2.5–80 μM for both ligustilide and 6-gingerol.

### 2.4. Effects of AZ-SFE and Major Components on NO Production in LPS-Induced RAW264.7 Cells

**Figure 3:**
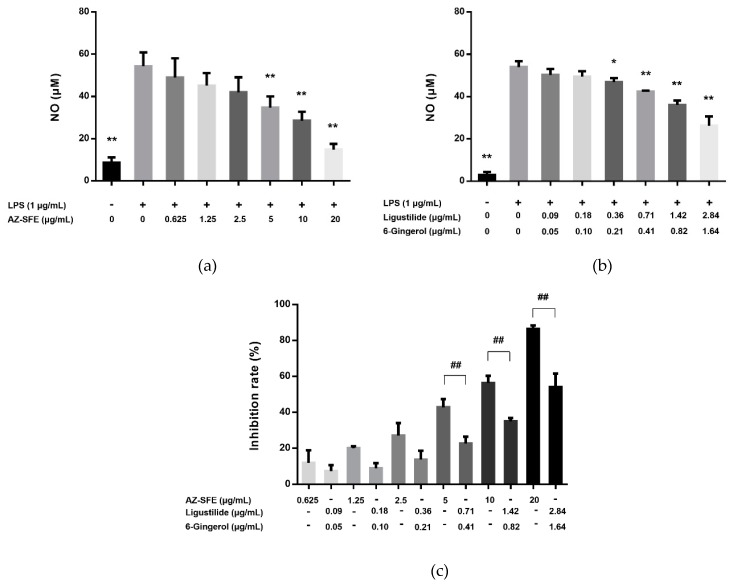
Effects of AZ-SFE and a combination of ligustilide and 6-gingerol on NO production in lipopolysaccharide (LPS)-induced RAW264.7 cells. Cells were pretreated for 1 h in the presence or absence of (**a**) AZ-SFE or (**b**) the combination of ligustilide and 6-gingerol, in a ratio according to their content in AZ-SFE, and then stimulated with LPS (1 μg/mL) for 24 h. (**c**) The inhibition rate was calculated by the formula indicated in Section 4.7. Data are expressed as mean ± SD (*n* = 3) of three independent experiments. * *p* < 0.05 and ** *p* < 0.01 versus LPS group. ## *p* < 0.01 between two compared groups.

LPS significantly induced the production of NO compared with the blank control. Gradient concentrations of AZ-SFE could reduce the level of NO in a dose-dependent manner (Figure 3a). Moreover, the combination of two major components, ligustilide and 6-gingerol, according to their proportion in AZ-SFE, suppressed the production of NO with the same trend as AZ-SFE, but more moderately (Figure 3b). On one hand, they contributed to more than half of the inhibitory effect at the highest dose (54.08% vs 86.37%) with less than half of the content of AZ-SFE, indicating their predominant effects. On the other hand, the inhibition rates between AZ-SFE and combination of ligustilide and 6-gingerol were significantly different, indicating that other compounds also took part in the suppression of NO production, which should not be ignored (Figure 3c). Therefore, the total AZ-SFE instead of a combination of ligustilide and 6-gingerol was used for in vivo study.

### 2.5. Effects of AZ-SFE on Splenocyte Proliferation and Cytokine Secretion

**Figure 4:**
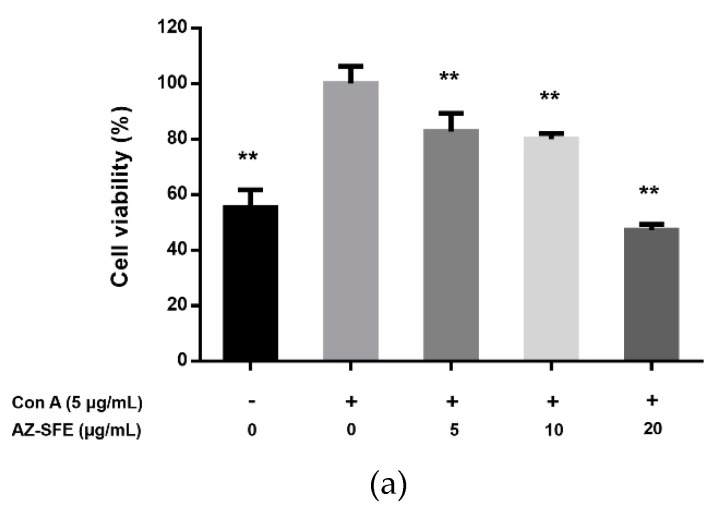

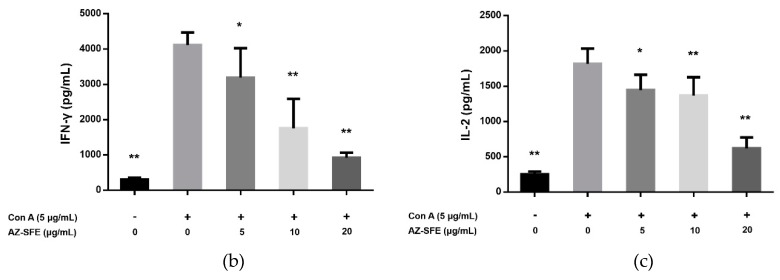
Effects of AZ-SFE (5, 10 and 20 μg/mL) on splenocyte proliferation and cytokine production. Cells treated without Con A and AZ-SFE were the blank control group, and cells incubated only with Con A were the Con A group. (**a**) Cell viability was detected by MTT assay, (**b**) IFN-γ and (**c**) IL-2 in supernatants were measured by ELISA. Data are presented as mean ± SD (*n* = 6). * *p* < 0.05, ** *p* < 0.01 versus Con A group.

With the stimulation of 5 μg/mL of Concanavalin A (Con A), splenocytes of rats in the TNBS group were obviously proliferated ex vivo compared with blank control. Treatment with SFE (5, 10 and 20 μg/mL) could dose-dependently and significantly inhibit the Con A-induced proliferation (Figure 4a). Also, the levels of IFN-γ and IL-2 in supernatants were decreased by administration of AZ-SFE (Figure 4b,c).

### 2.6. Effects of AZ-SFE on Body Weight and Disease Activity Index

**Figure 5:**
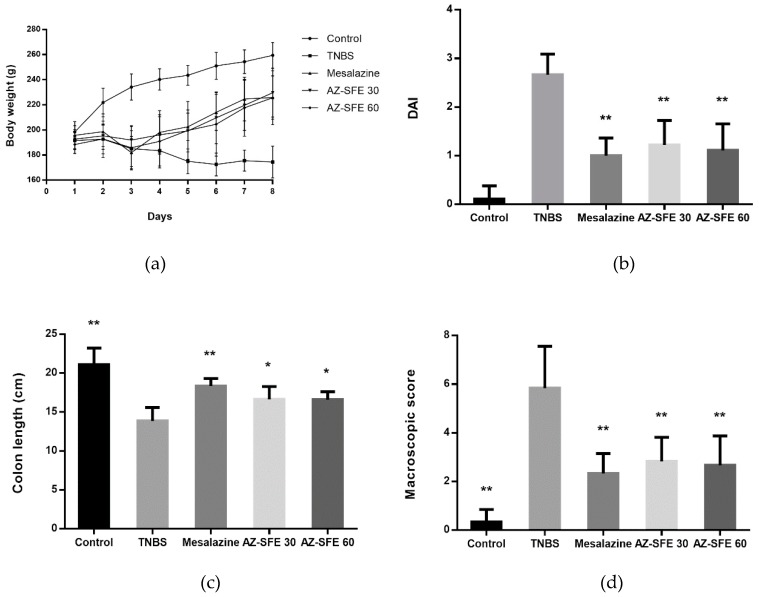
Effects of AZ-SFE (30 and 60 mg/kg) and mesalazine (400 mg/kg) on (**a**) body weight change, (**b**) disease activity index, (**c**) length of colons, and (**d**) macroscopic score of colons in TNBS-induced colitis rat model. Data are presented as mean ± SD of 6 rats per group. * *p* < 0.05, and ** *p* < 0.01 versus TNBS group.

Rats treated by TNBS presented diarrhea the day after modelling (Day two), with some of them displayed obvious blood adhesion to the anus. In the TNBS group, the body weights were dropped through experiment period accompanied with a loss of appetite, mucous or loose stools, and fecal occult blood. After treatment with AZ-SFE and mesalazine, the body weight of rats recovered obviously (Figure 5a). Also, the issues of stool consistency and fecal blood were improved in all treatment groups. As indicated in Figure 5b, the disease activity index (DAI) score on Day eight in the TNBS group was significantly higher than that in control group. And the DAI scores in AZ-SFE and mesalazine groups were significantly lower than that in TNBS group. The macroscopic appearances of colon in different groups were shown in Figure S1.

### 2.7. Effects of AZ-SFE on Colon Length and Macroscopic Score

The colon length of each rat was measured before cutting longitudinally, and the results showed that the colons in TNBS group became shorter than those in control group. Treatment with AZ-SFE (30 and 60 mg/kg) markedly relieved the shortening of the colon. The macroscopic changes were observed and compared among all groups. The colons of TNBS-treated rats displayed mucosal edema, hyperemia, colonic wall thickening, ulceration, necrosis, and tissue adhesion, while colons in control group were healthy with normal appearances. The macroscopic scores in TNBS group were significantly increased compared to the control group. Treatment with mesalazine and two doses of AZ-SFE markedly reduced the macroscopic lesions in colonic tissue, resulting in significantly decreased scores. The colon length and macroscopic score were shown in Figure 5c,d, respectively.

### 2.8. Effects of AZ-SFE on Histopathology Improvement

**Figure 6:**
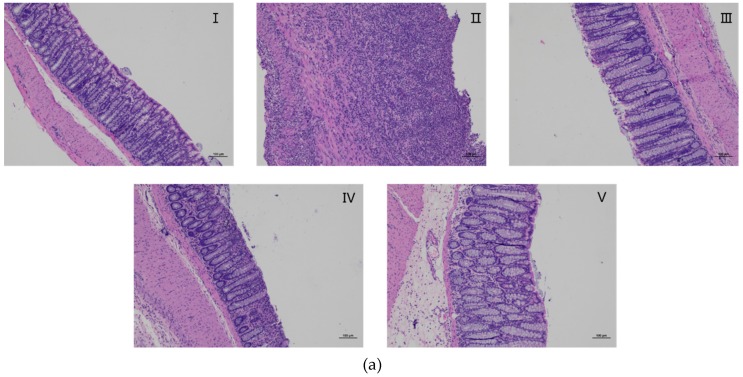

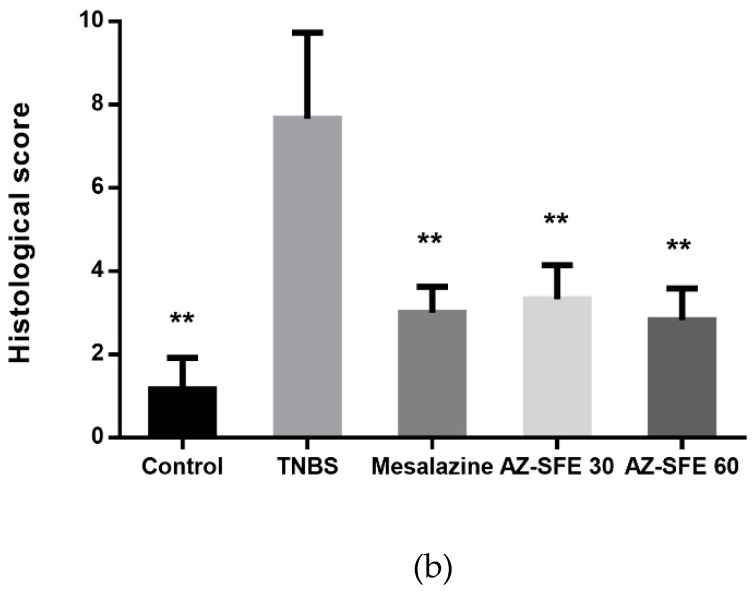
Effects of AZ-SFE (30 and 60 mg/kg) and mesalazine (400 mg/kg) on histological change of TNBS-induced colitis in rats. (**a**) Representative H&E staining slices from colonic tissues, original magnification 100× I. Control group; II. TNBS group; III. Mesalazine group; IV–V. AZ-SFE groups (30 and 60 mg/kg). (**b**) Histological scores. Data are presented as mean ± SD of 6 rats per group. ** *p* < 0.01 versus TNBS group.

Hematoxylin and eosin (H&E) stained colonic tissue specimens were observed under microscope to analyze histological features (Figure 6a). In the control group, the colons showed intact mucosa, and clear crypt structure with adequate goblet cells. However, the TNBS group displayed severe infiltration of inflammatory cells, deformed or disappeared crypt, ulcers, and thickened blood vessel walls, representing a high level of histological damage. The samples in AZ-SFE and mesalazine groups indicated progressive amelioration of pathological states with reduction of inflammatory infiltration and significant lower histological scores compared with TNBS group (Figure 6b).

### 2.9. Effects of AZ-SFE on superoxide dismutase (SOD), malonic dialdehyde (MDA) and myeloperoxidase (MPO)

**Figure 7:**
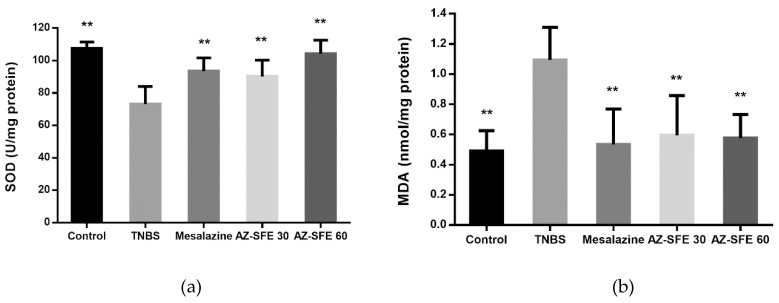

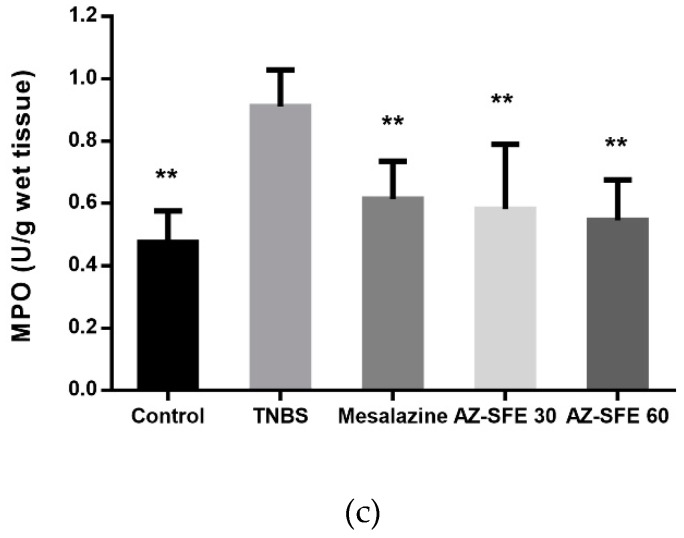
Effects of AZ-SFE (30 and 60 mg/kg) and mesalazine (400 mg/kg) on oxidative stress indicators in colons of TNBS-induced colitis in rats. (**a**) Superoxide dismutase (SOD). (**b**) Malonic dialdehyde (MDA). (**c**) Myeloperoxidase (MPO). Data are presented as mean ± SD of 6 rats per group. ** *p* < 0.01 versus TNBS group.

As shown in Figure 7, TNBS administration significantly reduced the activity of superoxide dismutase (SOD), which was recovered with the intervention of AZ-SFE and mesalazine. The MDA levels in the colons of the TNBS group significantly increased in comparison with control group and decreased after treatment with AZ-SFE and mesalazine. The myeloperoxidase (MPO) activity in TNBS group significantly increased compared with that in control group. After the treatment of AZ-SFE and mesalazine, the MPO levels were decreased.

### 2.10. Effects of AZ-SFE on Inflammatory Cytokines in Serum

**Figure 8:**
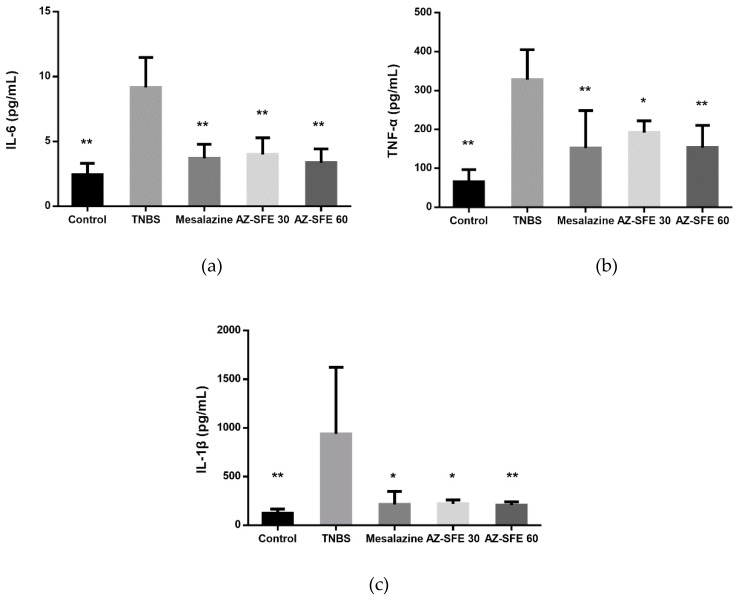
Effects of AZ-SFE (30 and 60 mg/kg) and mesalazine (400 mg/kg) on production of pro-inflammatory cytokines in serum of TNBS-induced colitis in rats. (**a**) IL-6. (**b**) TNF-α. (**c**) IL-1β. Data are presented as mean ± SD of 6 rats per group. * *p* < 0.05 and ** *p* < 0.01 versus TNBS group.

The levels of inflammatory cytokines (IL-6, TNF-α and IL-1β) were measured by ELISA. As shown in Figure 8, TNBS treatment significantly enhanced the production of cytokines compared with control group. Administration with two doses of AZ-SFE or mesalazine markedly inhibited the accumulations of three tested cytokines.

### 2.11. Effects of AZ-SFE on Serum Hepcidin and Serum Iron

**Figure 9:**
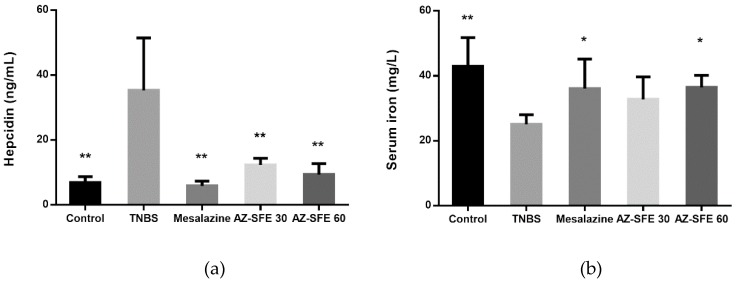
Effects of AZ-SFE (30 and 60 mg/kg) and mesalazine (400 mg/kg) on the levels of (**a**) serum hepcidin and (**b**) serum iron of TNBS-induced colitis in rats. Data are presented as mean ± SD of 6 rats per group. * *p* < 0.05 and ** *p* < 0.01 versus TNBS group.

Hepcidin levels in serum were increased in TNBS treated rats compared with the control group, which were correlated with the similar tendency of IL-6, TNF-α, and IL-1β. However, treatment with AZ-SFE (30 and 60 mg/kg) significantly decreased the high expression of hepcidin, and the effect of AZ-SFE at 60 mg/kg was better than that of AZ-SFE at 30 mg/kg (Figure 9a). The serum iron was measured by chemical colorimetry. As indicated in Figure 9b, the concentration of serum iron in TNBS group was significantly lower than that in control group. After intervention by AZ-SFE or mesalazine, concentrations of serum iron were increased. Particularly, mesalazine and AZ-SFE (60 mg/kg) groups showed significant difference in comparison with TNBS group.

## 3. Discussion

Natural products from medicinal and edible plants are supposed to be alternative treatments for chronic inflammatory diseases [24,25]. The multiple active constituents in plants are considered to act simultaneously on different targets to exert therapeutic effect [23]. In the present study, we optimized the extraction process of AZ-SFE, and analyzed the extract both qualitatively and quantitatively by GC/MS and HPLC methods, respectively. Subsequently, the obtained AZ-SFE with a stable quality was applied to the ensuing bioactivity evaluation.

Macrophages as important immune cells play a vital role in the inflammation response. Shin et al. found that macrophages could be accumulated and activated in dextran sodium sulphate (DSS)-induced colonic tissues [26]. Activated macrophages can secrete many kinds of inflammatory mediators, resulting in the progression of inflammatory diseases. LPS-induced RAW264.7 macrophages are classically used to evaluate the anti-inflammatory effect in vitro. NO, a pro-inflammatory mediator synthesized by iNOS, can be overproduced under the stimulation of LPS. Therefore, the inhibitory ability of AZ-SFE on NO production was assessed to preliminarily evaluate the anti-inflammatory effect. Results proved the anti-inflammatory activity of AZ-SFE, because of the significant and dose-dependent suppression of NO production. The in vitro study encouraged us to further investigate the anti-colitis activity of AZ-SFE on TNBS-induced rat model.

Immunological abnormity is one of the factors involving the pathogenesis of IBD [27]. Immunosuppressors like azathioprine have been applied for the treatment of moderate to severe patients [6]. The spleen is an important immune organ, containing a large number of T lymphocytes which can be non-specifically stimulated by Con A. IFN-γ can induce macrophages to produce TNF-α, IL-1β, IL-2, and IL-6, which together aggravate inflammation and magnify Th1 response in a positive feedback regulation [7]. The proliferation of ex vivo splenocytes induced by Con A was suppressed by AZ-SFE. Additionally, the levels of IFN-γ and IL-2 in cell cultures were reduced by AZ-SFE in a dose-dependent manner. Based on our findings, it could be speculated that the immunoregulating effect of AZ-SFE is related to the modulation of Th1 response, and the anti-colitis mechanism of AZ-SFE involves immunoregulation.

In vivo study revealed an anti-inflammatory effect of AZ-SFE in TNBS-induced rats with the alleviation of disease activity and macroscopic damage. Furthermore, histological evaluation supported the reduction of pathological changes by AZ-SFE intervention. It has been widely accepted that oxidative stress is one of the important pathogenic factors involving the development of IBD [28]. Oxidative stress and the resulting lipid peroxidation could exacerbate free radical chain reactions, disrupt the integrity of intestinal mucosa, and activate inflammatory mediators [29]. SOD is an important antioxidant enzyme which is able to scavenge free radicals, and hinder oxidative damage [30]. MDA is a major product of lipid peroxidation as a consequence of oxidative stress [31]. Our results showed that AZ-SFE increased SOD activity and decreased MDA concentration in colonic tissue of TNBS-induced rats, indicating the amelioration of intestinal injury was at least partly related to the reduction of oxidative stress. Neutrophil infiltration is one of the most prominent histological features observed in IBD [29]. MPO activity can be used to evaluate the degree of neutrophil infiltration [32,33]. Our findings revealed that increased MPO activity induced by TNBS could be weakened after administration of AZ-SFE at test doses, showing the protective effect of AZ-SFE against tissue injury in experimental colitis.

Pro-inflammatory cytokines are important in the pathogenesis of IBD. Increased levels of pro-inflammatory cytokines have been detected in IBD patients [34,35]. TNF-α takes part in tissue inflammation through recruitment of leukocytes in an inflamed area, stimulation of expression of cytokines, and induction of cascade effects for other cytokines [36]. IL-6 also plays a vital role in the progression of colonic inflammation, which can promote lymphocyte proliferation, and is important in acute phase inflammation response [36,37]. IL-1β is another important cytokine to accelerate intestinal inflammation by facilitating the production of IL-17A, indicating IL-1β to be a promising target in IBD therapy [38]. Taken together, targeting pro-inflammatory cytokines is one of the therapeutic approaches for IBD treatment. In the current study, the production of IL-6, TNF-α, and IL-1β in serum of TNBS-induced rats was increased. Treatment with AZ-SFE in two doses significantly reduced the expression of tested cytokines, with the dose of 60 mg/kg showing the better effect.

Anemia is one of the symptoms of IBD, whose pathogenesis is related to the abnormal elevation of hepcidin, the key modulator of systemic iron homeostasis [39]. Hepcidin is an antimicrobial peptide which is mainly generated in liver in response to iron overload, or upregulation by pro-inflammatory stimuli, such as IL-6 [40]. The cellular iron export protein ferroportin is the receptor of hepcidin. The combination of hepcidin and ferroportin contributes to the reduction of circulating iron. Therefore, it is supposed that increased level of hepcidin leads to decreased serum iron, consequentially iron-restricted impairment of erythropoiesis, and even anemia [41]. Many researchers have been interested in finding the correlation between hepcidin and IBD, which up till now has not been fully clarified. Some reported the elevated expression of hepcidin in IBD patients, while others claimed no differences or even decreased hepcidin levels compared with healthy controls [42,43,44]. In the TNBS-induced rat colitis model, the expression of hepcidin in the colon could be increased associated with the activation of IL-6/STAT 3 pathway [45]. Also, hepcidin has been proposed to be directly involved in IBD pathogenesis, since the severity of experimental colitis could be relieved in *Hfe* knockout mice with low hepcidin expression [46]. Taken together, we intended to investigate the effect of AZ-SFE on the regulation of hepcidin in our study.

To some extent, our results support the explanation that the higher hepcidin level in TNBS group is the consequence of the more severe inflammation state, as Toblli et al. described [47]. Administration of AZ-SFE and mesalazine decreased the production of hepcidin, indicating the potency of AZ-SFE on the regulation of iron homeostasis. The expression of hepcidin was related to the production of IL-6, as shown in our research, which was consistent with former research. Also, the serum iron level was decreased in the TNBS group, while it was significantly increased by the administration of mesalazine and AZ-SFE (60 mg/kg). On one hand, the increased expression of hepcidin lead to the decrease of serum iron [41]. On the other hand, infiltrating cells may enter the blood and utilize serum iron for their proliferation, and the sequestration of serum iron by iron storage proteins may lead to the low level of serum iron [48]. These findings indicated that AZ-SFE could involve in the regulation of system iron homeostasis, which may be related to its anti-inflammatory activity.

Furthermore, intestinal epithelial cells provide a physical barrier to protect the body from pathogens as well as toxins [49]. The dysfunction of intestinal barrier, such as increased intestinal permeability plays an important role in the pathogenesis of IBD. Therefore, the protection and improvement of intestinal barrier function may be a potential therapeutic strategy for IBD [50]. The effects of AZ-SFE on the intestinal barrier will be investigated in our further research.

## 4. Materials and Methods

### 4.1. Chemicals and Reagents

Ligustilide (S13M9D55861) was obtained from Shanghai Yuanye Biotechnology Co., Ltd., (Shanghai, China). 6-gingerol (MUST-16122205) was purchased from Chengdu Man Site Biotechnology Co. Ltd., (Chengdu, China). Methanol and Acetonitrile were HPLC grade (Sigma-Aldrich, USA). High glucose DMEM was purchased from Corning Incorporated (Corning, NY, USA). Fetal bovine serum (FBS) was obtained from Biological Industries Ltd. (Herzliya, Israel). Penicilin/streptomycin was obtained from Invitrogen (Carlsbad, CA, USA). TNBS (P2297–10 mL) was purchased from Sigma-Aldrich (St. Louis, MO, USA). Mesalazine was obtained from Ethypharm Pharmaceutical Co. Ltd., (Shanghai, China). All other reagents used were of analytical grade.

### 4.2. Plant Material and Preparation of AZ-SFE

Fresh *Zingiber officinale* Roscoe roots were bought in a local market (Beijing, China). *Angelica sinensis* was purchased from Beijing Sanhe Yaoye Co., Ltd. (Beijing, China). They were identified at the Beijing University of Chinese Medicine as per the identification standard of Pharmacopoeia of the People’s Republic of China, 2015. Fresh *Zingiber officinalle* Roscoe roots were cut into slices and dried at 40 °C. Then they were smashed into 40 mesh. Extraction was conducted using a HA220-50-06 supercritical fluid extraction system (Hua’an Supercritical Extraction Co., Ltd. Nantong, China). Ground powders (40 mesh) of *Angelica sinensis* and *Zingiber officinalle* Roscoe were weighted and extracted together at a ratio of 7:4 which is based on a traditional usage. When the temperatures in both extraction and separation vessels met the requirement, liquid CO_2_ was pumped into the extraction system at a flow rate of 25 L/h. The pressure of first separation vessel was 8 MPa; the temperature of first separation vessel was 55 °C; while those of second separation vessel were system tail pressure and 35 °C. After extraction, the products were collected from the first separation vessel, weighted and stored at −20 °C for further analysis.

### 4.3. GC/MS Analysis of AZ-SFE

GC/MS analysis of AZ-SFE was carried out on Agilent 7890B GC system coupled with 5977A mass selective detector (Agilent Technologies Inc., Santa Clara, CA, USA) in electronic ionization mode (ionization energy: 70 eV). The GC column was Agilent HP-5ms (30 m × 0.25 mm, 0.25 μm). AZ-SFE was dissolved in chloroform for analysis. The heating temperature was as follows: Hold at 50 °C for 5 min, rise to 170 °C at the rate of 10 °C/min, and hold for 5 min; then rise to 230 °C at the rate of 3 °C/min and hold for 3 min; finally rise to 280 °C at the rate of 5 °C/min and hold for 5 min. Inlet temperature and transmission line temperature were both 250 °C. Helium was used as carrier gas at a flow rate of 1 mL/min. The injection volume was 1 μL with a split ratio of 10:1. Ion source temperature was 230 °C and quadrupole the temperature was at 150 °C. The scan scale was 30–600 amu.

### 4.4. HPLC Analysis of AZ-SFE

The contents of ligustilide and 6-gingerol in AZ-SFE were detected by HPLC in a Thermo Ultimate 3000 HPLC system with a variable wavelength detector (Thermo Fisher Scientific Inc., San Francisco, CA, USA) using an Inertsil ODS-C18 column (4.6 mm × 250 mm, 5 μm). The column temperature was 30 °C, and 10 μL of the sample solution was injected for detection. AZ-SFE, ligustilide and 6-gingerol were dissolved in methanol as a sample solution and reference solution, respectively. The flow rate was 1 mL/min. For detection of ligustilide, the mobile phase was methanol-water (70:30), and the detection wavelength was 326 nm. For detection of 6-gingerol, the mobile phase was acetonitrile-methanol-water (40:5:55), and the detection wavelength was 280 nm [51]. The specificity, linearity, precision, repeatability, stability, and recovery rate of analytical methods were validated.

### 4.5. Optimization of Extraction Process of AZ-SFE

An L_9_ (3^4^) orthogonal experiment design was conducted to investigate the optimum parameters in the extraction process. The factors and levels were listed in Table 4 according to pre-experiments and literature review. The extract yield, ligustilide content and 6-gingerol content were chosen as evaluation indexes with weight coefficients of 0.5, 0.3, and 0.2, respectively. The colligation score of each extraction was calculated based on weight coefficient after data normalization. Optimum extraction parameters were screened by both visual analysis and analysis of variance by SAS 8.0.

### 4.6. Cell Culture and MTT Cell Viability Assay

The RAW264.7 cell line was obtained from the Cell Resource Center, Chinese Academy of Medical Sciences (Beijing, China). RAW264.7 cells were cultured in high glucose DMEM supplemented with 10% FBS and 1% penicillin/streptomycin (complete medium) at 37 °C, 5% CO_2_. AZ-SFE was dissolved in DMSO to be 10 mg/mL as stock solution. MTT cell viability assay was conducted at first to select concentrations without cytotoxicity based on the method of Mosmann with minor modifications [52]. RAW264.7 cells were seeded in 96-well plates (1 × 10^4^ cells/well) and cultured for 24 h. Then cells were incubated with gradient concentrations of AZ-SFE (0.625 to 40 μg/mL), ligustilide (2.5 to 160 μM), and 6-gingerol (2.5 to 160 μM) for 24 h. Then 10 μL of MTT solution (5 mg/mL) was added to each well and the cells were cultured for another 4 h. The supernatants were discarded and the formazan crystals formed in living cells were dissolved in DMSO. The plate was shaken in orbital shaker for 10 min, after which the optical density was measured at 490 nm using a microplate reader system (SPECTROstar Nano, BMG LABTECH, Ortenberg, Germany). The medium group contained only complete medium, and vehicle group contained 0.5% DMSO in complete medium. The cell viability was calculated as follows:

Viability% = OD/OD_0_ × 100% (1)

Where OD represents the average optical density of samples at the same concentration, and OD_0_ represents for the average optical density of vehicle controls (0.5% DMSO in medium).

### 4.7. Measurement of NO Production in RAW264.7 Cells

RAW264.7 cells were seeded into 96-well plates (1 × 10^5^ cells/well) and precultured for 24 h at 37 °C, 5% CO_2_. After discarding the supernatants, cells were treated with different concentrations of AZ-SFE (0.625 to 20 μg/mL) or the combinations of ligustilide and 6-gingerol, at their corresponding concentrations in AZ-SFE for 1 h. After stimulation with or without LPS (1 μg/mL) for 24 h, the cell supernatants were collected and detected for NO production by Griess method using a commercial kit (Beijing BioDee Biotechnology Co. Ltd., Beijing, China) [53]. Briefly, 50 μL of supernatant was mixed with the same volume of Griess reagent I and II. After incubation at room temperature for 5 min, the optical density was measured at 540 nm. The concentration of NO was calculated by comparing with sodium nitrite standard curve. The inhibition rate of NO was calculated as follows:

Inhibition rate% = (C_1_ − C)/(C_1_ − C_0_) (2)

Where C_1_ represents the average concentration of model controls (1 μg/mL LPS in medium), C_0_ represents the average concentration of blank control and C represents the average concentration of samples at the same concentration.

### 4.8. Experimental Animals

Thirty male Sprague Dawley rats (180–200 g) were purchased from SPF Biotechnology Co., Ltd. (Beijing, China) and maintained in the animal experiment center of Beijing University of Chinese medicine. Animals were housed under controlled conditions of temperature (25 ± 2 °C) and humidity (50 ± 10%) with a 12-h light-dark cycle, and fed with standard diet and water. The experimental procedures were approved by the Animal Care and Research Ethics Committee of the Beijing University of Chinese Medicine.

### 4.9. Induction of Experimental Colitis and Intervention with AZ-SFE

Rats were randomly divided into 5 groups (*n* = 6): Control group, TNBS group, positive drug (mesalazine, 400 mg/kg) group, and AZ-SFE (30 and 60 mg/kg) groups. Colitis was induced by intrarectal administration of a single dose of TNBS, as described previously, with modifications [54]. Briefly, rats were anesthetized by 10% chloral hydrate (3 mL/kg), and TNBS (100 mg/kg) dissolved in ethanol (50%, *v/v*) was instilled into the colon, using a gavage needle lubricated by liquid paraffin, and inserted into 8 cm from anus. To ensure the agent within the entire colon and avoid drug leakage, rats were held in a head-down position for 3 min. Control group received only saline by the same method.

From the next day after modeling, drugs were administered once a day by gavage (1 mL/100 g body weight) for seven consecutive days. Then the rats were anesthetized and blood were collected from abdominal aorta. The entire colons were collected, and washed in cold saline. All specimens were stored at −80 °C before analysis.

### 4.10. Splenocyte Proliferation and Cytokine Detection

The spleens of rats in the TNBS group were aseptically taken out, and they were ground with PBS and filtered through 200 mesh. After removing red blood cells by lysis and centrifugation, splenocytes were obtained. Cells were precultured in RPMI 1640 complete medium (supplemented with 10% FBS and 1% penicillin/streptomycin) for 2 h to remove adherent cells. Then cells in suspension were counted and seeded into 96-well plates (5 × 10^5^ cells/well). Cells were stimulated with 5 μg/mL Con A to maintain inflammation status, and AZ-SFE (5, 10 and 20 μg/mL) were supplemented and incubated for 24 h. Cells treated with Con A were named the Con A group, and cells without any intervention were labelled blank control group. After incubation, culture medium in each well was collected and centrifuged. Supernatants were collected for cytokine detection, and cells were cultured with MTT in medium for another 4 h, for proliferation detection. The optical density was measured at 550 nm. The levels of IL-2 and IFN-γ in supernatants were measured using ELISA kits (RayBiotech Inc., Norcross, GA, USA) according to the manufacturer’s instructions.

### 4.11. Evaluation of DAI

Body weight, stool consistency and fecal bleeding of rats were monitored daily, and the DAI was calculated as the average score of three above mentioned aspects. In weight loss, score 0 was assigned for no weight loss compared with the original body weight, 1 for loss of 1–5%, 2 for loss of 5–10%, 3 for loss of 10–20% and 4 for loss > 20%. For stool consistency, score 0 was assigned for normal stool, 2 was assigned for loose stool and 4 was assigned for diarrhea. For fecal bleeding, score 0 represented no blood, 2 represented occult blood and 4 represented obvious bleeding [55].

### 4.12. Evaluation of Macroscopic Damage

The colon was cut longitudinally and cleaned by cold saline for macroscopic observation. The macroscopic severity of colonic mucosal damage was determined according to the criteria of Luk et al. [56]. Briefly, the score was on a 0–10 scale. The more severe the damage was, the higher the score was.

### 4.13. Histological Analysis

Colon samples were fixed in 10% formalin at room temperature for 48 h, and embedded in paraffin blocks. Sliced sections (3 μm) were deparaffinized and stained with H&E. The sections were observed and photographed under an ECLIPSE Ts2R microscope (Nikon Corp., Tokyo, Japan). Colonic damage was evaluated according to previous standard [57] with a total score of 10.

### 4.14. Measurement of SOD, MDA and MPO in Colonic Tissue

Colonic tissues were homogenized, and the supernatants were collected for detection. The activities of SOD and MPO, as well as the level of MDA were analyzed with commercial test kits (Nanjing Jiancheng Bioengineering Institute, Nanjing, China).

### 4.15. Measurement of Pro-inflammatory Cytokines in Serum

Blood samples collected from abdominal aorta were placed at room temperature for 2 h to clot, and they were centrifuged at 3000 rpm for 10 min in order to collect serum. The levels of IL-6, TNF-α and IL-1β in serum were detected using ELISA kits, according to the manufacturer’s instructions. The kit for IL-6 was purchased from Cloud Clone Corp., (Wuhan, China), and those for TNF-α and IL-1β were obtained from RayBiotech Inc. (Norcross, GA, USA).

### 4.16. Measurement of Hepcidin and Serum Iron

Serum hepcidin was measured by competitive ELISA (Cloud Clone Corp., Wuhan, China). Concentrations of serum iron were detected by colorimetry using a test kit (Nanjing Jiancheng Bioengineering Institute, Nanjing, China).

### 4.17. Statistic Analysis

Data obtained were presented as the mean ± standard deviation (SD). Statistical analyses were performed using one-way ANOVA, followed by Dunnett’s multiple comparisons tests for multiple comparisons using in SAS 8.0. *p* < 0.05 was considered statistically significant.

## 5. Conclusions

Our present study prepared and characterized AZ-SFE. The ensuing in vitro and in vivo study indicated the potential of AZ-SFE for relieving colitis by decreasing oxidative stress, suppressing inflammatory mediators, inhibiting the Th1 immune response, and regulating iron homeostasis. In conclusion, AZ-SFE derived from traditional Chinese herbs could be a promising supplement for current IBD therapy, and the exact mechanism needs further investigation.

## Figures and Tables

**Table 1:** Qualitative analysis of *Angelica sinensis* and *Zingiber officinale* Roscoe’s supercritical fluid extract (AZ-SFE) based on GC/MS analysis.

No.	Retention Time (min)	Compound Name	Molecular Formula
1	13.211	Decanal	C_10_H_20_O
2	17.137	α-Curcumene	C_15_H_22_
3	17.202	β-Copaene	C_15_H_2__4_
4	17.305	Zingiberene	C_15_H_2__4_
5	17.422	α-Farnesene	C_15_H_2__4_
6	17.480	β-Bisabolene	C_15_H_2__4_
7	17.706	β-Sesquiphellandrene	C_15_H_2__4_
8	18.114	Hedycaryol	C_15_H_2__6_O
9	19.129	Zingiberenol	C_15_H_2__6_O
10	19.770	Zingerone	C_11_H_14_O_3_
11	19.930	N-Butylphthalide	C_12_H_14_O_2_
12	20.003	β-Eudesmol	C_15_H_26_O
13	20.378	N-Butylidenephthalide	C_12_H_12_O_2_
14	20.824	Dehydronerolidol	C_15_H_24_O
15	21.54	Senkyunolide	C_12_H_16_O_2_
16	22.060	Z-Ligustilide	C_12_H_14_O_2_
17	23.657	E-Ligustilide	C_12_H_14_O_2_
18	28.392	Hexadecanoic acid	C_16_H_32_O_2_
19	29.718	Senkyunolide H	C_12_H_16_O_4_
20	33.626	Linoleic acid	C_18_H_32_O_2_
21	35.346	Panaxynone	C_17_H_22_O
22	36.219	6-Paradol	C_17_H_26_O_3_
23	37.934	6-Shogaol	C_17_H_24_O_3_
24	38.917	6-Gingerdione	C_17_H_24_O_4_
25	40.638	6-Gingerol	C_17_H_26_O_4_
26	42.513	6-Gingerol monoacetate	C_19_H_28_O_5_
27	43.613	8-Shogaol	C_19_H_30_O_4_
28	44.435	6-Gingerdiol 3,5-diacetate	C_21_H_32_O_6_
29	44.758	8-Gingerdione	C_19_H_28_O_4_
30	46.518	6-Dehydrogingerdione	C_17_H_22_O_4_
31	46.731	8-Gingerol	C_19_H_30_O_4_
32	49.500	10-Shogaol	C_21_H_32_O_3_
33	50.353	10-Gingerdione	C_21_H_32_O_4_
34	55.218	10-Dehydrogingerdione	C_21_H_30_O_4_

**Table 2:** Results of orthogonal experimental design for extraction optimization of AZ-SFE. A represented extraction pressure, B represented extraction temperature, C represented extraction time and D was blank.

Run	Factor				Evaluation Index			
	A	B	C	D	Yield (%)	Ligustilide Content (%)	6-Gingerol Content (%)	Score
1	1	1	1	1	1.74	15.51	8.03	76.23
2	1	2	2	2	1.84	15.14	8.99	79.34
3	1	3	3	3	2.20	15.19	8.91	85.35
4	2	1	2	3	1.88	15.04	9.58	80.96
5	2	2	3	1	2.35	14.25	7.74	83.77
6	2	3	1	2	2.92	14.05	8.50	94.65
7	3	1	3	2	1.68	13.68	9.70	75.17
8	3	2	1	3	2.06	15.54	8.66	83.04
9	3	3	2	1	2.29	12.58	9.57	83.23
K1	80.31	77.45	84.64	81.08				
K2	86.46	82.05	81.18	83.05				
K3	80.48	87.74	81.43	83.12				
R	6.15	10.29	3.46	2.04				

**Table 3:** Variance analysis of orthogonal experimental design for extraction optimization of AZ-SFE. * *p* < 0.05.

Factor	DF	Anova SS	Mean Square	F	P
Pressure	2	73.65	36.83	9.12	0.0988
Temperature	2	159.43	79.71	19.75	0.0482 *
Time	2	22.36	11.18	2.77	0.2652
Error	2	8.07	4.04		

**Table 4:** Factors and levels for orthogonal experiment design for optimization of SFE extraction process.

Level	Factor		
	Pressure (MPa)	Temperature (°C)	Time (h)
	A	B	C
**1**	20	35	1
**2**	30	45	2
**3**	40	55	3

## Supplementary Materials

Supplementary materials can be found at https://www.mdpi.com/1422-0067/20/15/3816/s1.

## Abbreviations

AZ-SFE	Supercritical fluid extract of *Angelica sinensis* and *Zingiber officinale* Roscoe
Con A	Concanavalin A
CD	Crohn’s disease
DAI	Disease activity index
FBS	Fetal bovine serum
H&E	Hematoxylin and eosin
IBD	Inflammatory bowel disease
LPS	Lipopolysaccharide
MDA	Malonic dialdehyde
MPO	Myeloperoxidase
SOD	Superoxide dismutase
TNBS	2, 4, 6-Trinitrobenzenesulfonic acid
UC	Ulcerative colitis
