# Supplementary material for: Supercritical Fluid Extract of Angelica sinensis and Zingiber officinale Roscoe Ameliorates TNBS-Induced Colitis in Rats
Source: Int J Mol Sci. 2019 Aug 5;20(15):3816. doi: 10.3390/ijms20153816 (PMC6696010; doi:10.3390/ijms20153816)
Supplement: Supplementary file 1 [file ijms-20-03816-s001.zip › ijms-545462-supplementary.docx]

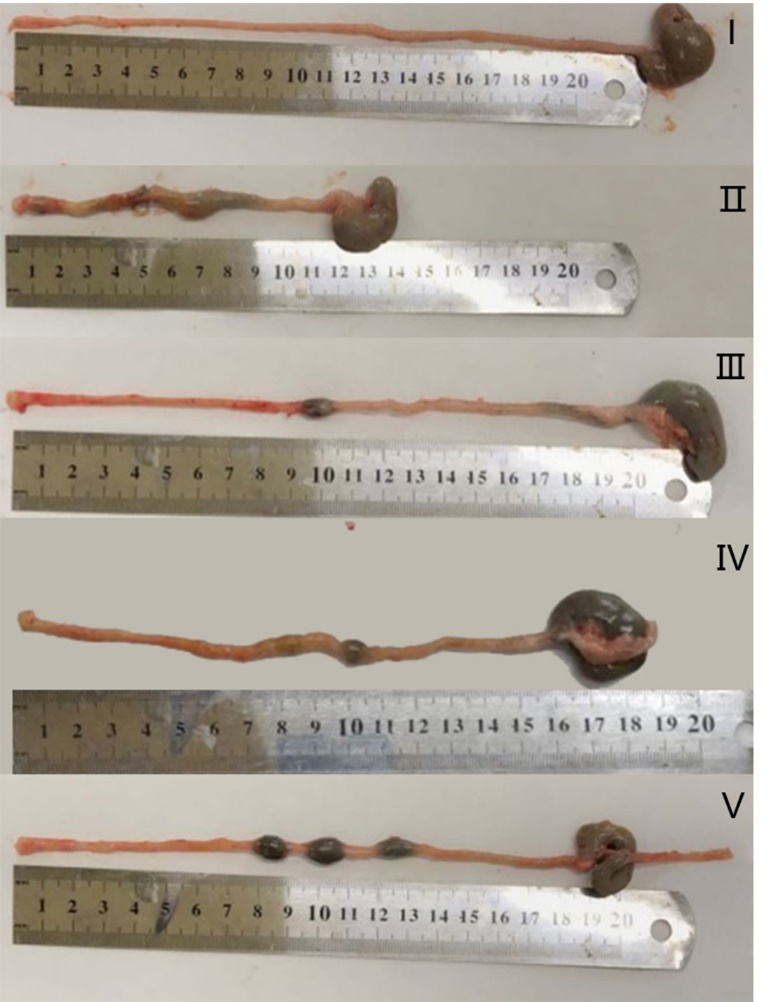


Figure S1. Macroscopic appearance of colon in different groups. Ⅰ represented colon in control group, Ⅱ represented colon in TNBS group, Ⅲ represented colon in mesalazine group, Ⅳ and Ⅴ represented colons in AZ-SFE groups (30 and 60 mg/kg).
